# Global Perspectives on Immunization During Pregnancy and Priorities for Future Research and Development: An International Consensus Statement

**DOI:** 10.3389/fimmu.2020.01282

**Published:** 2020-06-24

**Authors:** Bahaa Abu-Raya, Kirsten Maertens, Kathryn M. Edwards, Saad B. Omer, Janet A. Englund, Katie L. Flanagan, Matthew D. Snape, Gayatri Amirthalingam, Elke Leuridan, Pierre Van Damme, Vana Papaevangelou, Odile Launay, Ron Dagan, Magda Campins, Anna Franca Cavaliere, Tiziana Frusca, Sofia Guidi, Miguel O'Ryan, Ulrich Heininger, Tina Tan, Ahmed R. Alsuwaidi, Marco. A. Safadi, Luz M. Vilca, Nasamon Wanlapakorn, Shabir A. Madhi, Michelle L. Giles, Roman Prymula, Shamez Ladhani, Federico Martinón-Torres, Litjen Tan, Lessandra Michelin, Giovanni Scambia, Nicola Principi, Susanna Esposito

**Affiliations:** ^1^Department of Pediatrics, University of British Columbia, Vancouver, BC, Canada; ^2^Faculty of Medicine and Health Sciences, Centre for the Evaluation of Vaccination, Vaccine and Infectious Diseases Institute, University of Antwerp, Antwerp, Belgium; ^3^Division of Pediatric Infectious Diseases, Department of Pediatrics, Vanderbilt University School of Medicine, Nashville, TN, United States; ^4^Department of Internal Medicine (Infectious Diseases), Department of Epidemiology of Microbial Diseases, Yale School of Medicine, Yale School of Public Health, New Haven, CT, United States; ^5^Department of Pediatrics, Seattle Children's Research Institute, University of Washington, Seattle, WA, United States; ^6^Faculty of Health Sciences, School of Medicine, University of Tasmania, Launceston, TAS, Australia; ^7^School of Health and Biomedical Science, RMIT University, Melbourne, VIC, Australia; ^8^Department of Immunology and Pathology, Monash University, Melbourne, VIC, Australia; ^9^Oxford Vaccine Group, Department of Paediatrics, University of Oxford, Oxford, United Kingdom; ^10^Immunisation and Countermeasures Division, National Infection Service, Public Health England, London, United Kingdom; ^11^Third Department of Pediatrics, University Hospital ATTIKON, National and Kapodistrian University of Athens, Athens, Greece; ^12^Université de Paris, Inserm, CIC 1417, F-CRIN I REIVAC, Assistance Publique-Hôpitaux de Paris, Paris, France; ^13^The Faculty of Health Sciences, Ben-Gurion University of the Negev, Beer-Sheva, Israel; ^14^Preventive Medicine and Epidemiology Department, Hospital Universitario Vall d'Hebron, Barcelona, Spain; ^15^Dipartimento Scienze della Salute della Donna e del Bambino e di Sanità Pubblica, Fondazione Policlinico Universitario “A. Gemelli” IRCCS-Università Cattolica del Sacro Cuore, Rome, Italy; ^16^Department of Medicine and Surgery, Obstetrics and Gynaecology Unit, University of Parma, Parma, Italy; ^17^Microbiology and Mycology Program, Faculty of Medicine, Institute of Biomedical Sciences and Associate Researcher, Millennium Institute of Immunology and Immunotherapy, University of Chile, Santiago, Chile; ^18^Pediatric Infectious Diseases, University of Basel Children's Hospital, Basel, Switzerland; ^19^Division of Pediatric Infectious Diseases, Department of Pediatrics, Northwestern University Feinberg School of Medicine, Ann & Robert H. Lurie Children's Hospital of Chicago, Chicago, IL, United States; ^20^Department of Pediatrics, College of Medicine and Health Sciences, United Arab Emirates University, Al Ain, United Arab Emirates; ^21^Department of Pediatrics, Santa Casa de São Paulo School of Medical Sciences, São Paulo, Brazil; ^22^Unit of Obstetrics and Gynecology, Buzzi Hospital - ASST Fatebenefratelli Sacco, University of Milan, Milan, Italy; ^23^Center of Excellence in Clinical Virology, Department of Pediatrics, Faculty of Medicine, Chulalongkorn University, Bangkok, Thailand; ^24^Department of Science and Technology/National Research Foundation: Vaccine Preventable Diseases, Faculty of Health Sciences, University of the Witwatersrand, Johannesburg, South Africa; ^25^Medical Research Council: Respiratory and Meningeal Pathogens Research Unit, Faculty of Health Sciences, University of the Witwatersrand, Johannesburg, South Africa; ^26^Department of Obstetrics and Gynaecology, Monash University, Melbourne, VIC, Australia; ^27^School of Medicine Hradec Kralove, Institute of Social Medicine, Charles University Prague, Prague, Czechia; ^28^Translational Pediatrics and Infectious Diseases, Pediatrics Department, Hospital Clínico Universitario de Santiago de Compostela, University of Santiago, Santiago de Compostela, Spain; ^29^Immunization Action Coalition, St. Paul, MN, United States; ^30^Infectious Diseases and Vaccinology Division, Health Sciences Post Graduation Program, University of Caxias Do Sul, Caxias Do Sul, Brazil; ^31^Università degli Studi di Milano, Milan, Italy; ^32^Department of Medicine and Surgery, Pediatric Clinic, Pietro Barilla Children's Hospital, University of Parma, Parma, Italy

**Keywords:** group B *Streptococcus* vaccines, influenza, maternal immunization, pertussis, pregnant women, respiratory syncytial virus, tetanus

## Abstract

Immunization during pregnancy has been recommended in an increasing number of countries. The aim of this strategy is to protect pregnant women and infants from severe infectious disease, morbidity and mortality and is currently limited to tetanus, inactivated influenza, and pertussis-containing vaccines. There have been recent advancements in the development of vaccines designed primarily for use in pregnant women (respiratory syncytial virus and group B *Streptococcus* vaccines). Although there is increasing evidence to support vaccination in pregnancy, important gaps in knowledge still exist and need to be addressed by future studies. This collaborative consensus paper provides a review of the current literature on immunization during pregnancy and highlights the gaps in knowledge and a consensus of priorities for future research initiatives, in order to optimize protection for both the mother and the infant.

## Introduction

Vaccination of pregnant women induces a vaccine-specific immune response in the mothers and the transfer of vaccine-specific antibodies via the placenta and breastmilk to directly protect the infant during the first months of life from the targeted pathogens (1, 2). The potential of maternal immunization in protecting young infants was made evident by tetanus vaccination during pregnancy contributing to the reduction in incidence of neonatal tetanus (3). This has also become evident by the decrease in the incidence of severe pertussis disease in young infants in countries that have implemented pertussis immunization programs in pregnancy (4–7).

**Figure 1:**
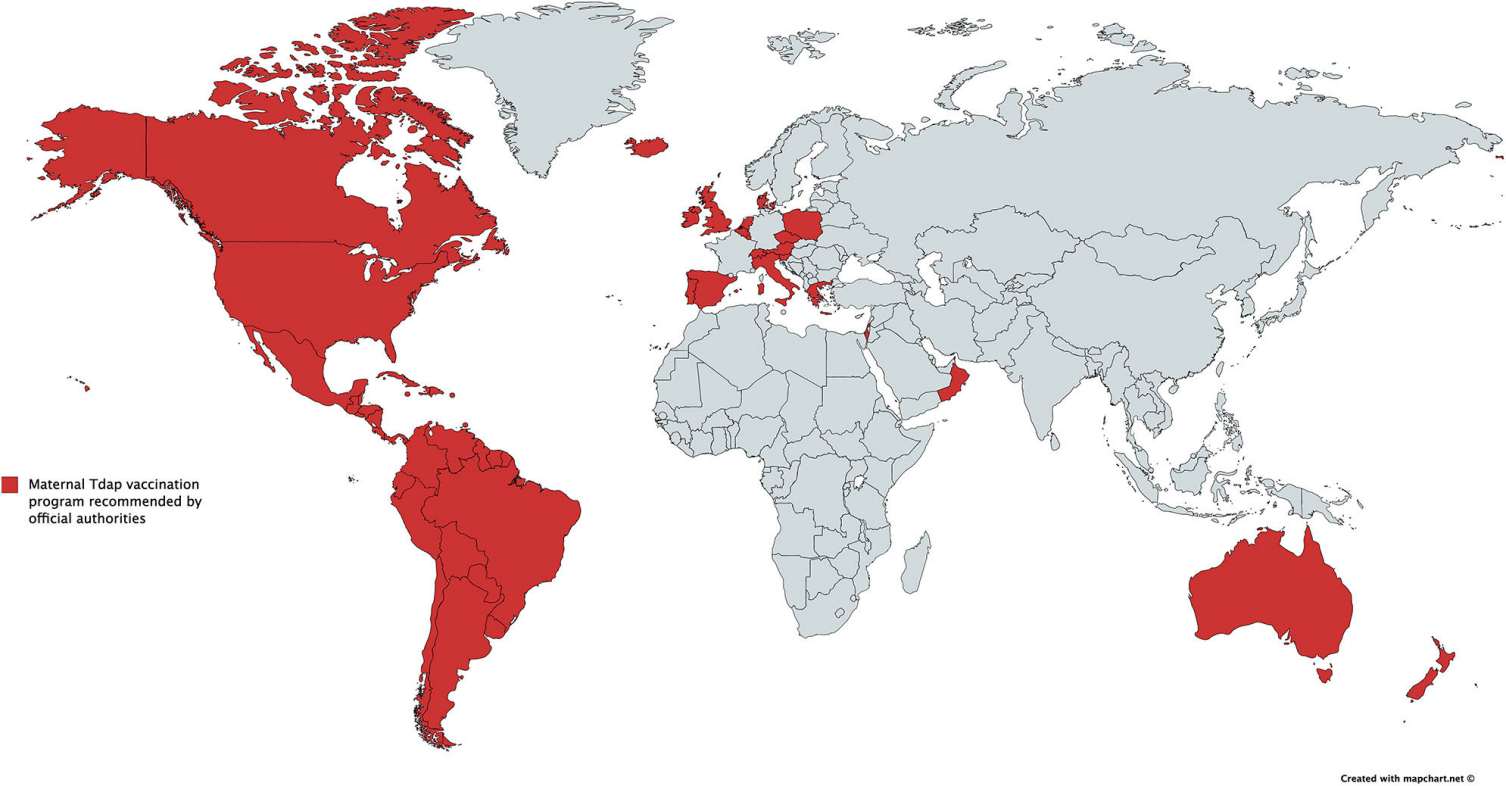
Countries with recommendations for immunization against pertussis in pregnancy by official authorities (for South America, pertussis immunization during pregnancy is recommended by The Pan American Health Organization). This figure was inspired by G. Amirthalingham and K. Maertens and created by K. Maertens.

During the last decade, an increasing number of countries have included vaccines for pregnant women in their national vaccination programs. Vaccination with tetanus-containing vaccines in pregnancy has been recommended for years in most low and middle -income countries (LMICs) (3), and pertussis and influenza vaccination programs for pregnant women have been more recently recommended in a number of high-income countries (HICs) and LMICs (Figure 1) (8, 9). Moreover, the prevention of respiratory syncytial virus (RSV) and group B *Streptococcus* (GBS) infections in infants through maternal vaccination has become a priority and a target for potential new vaccine candidates in trials and development (10–12).

**Figure 2:**
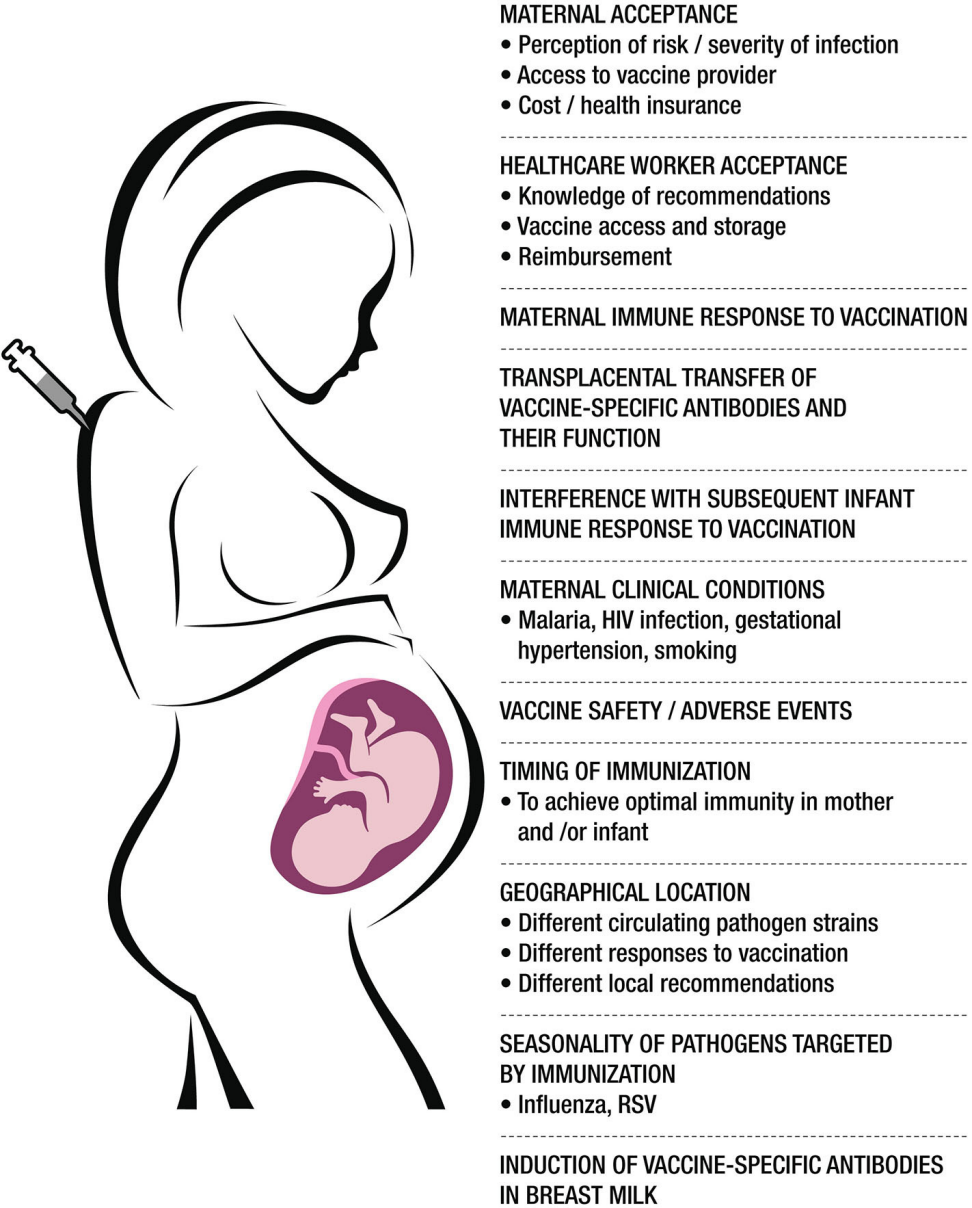
A summary of the major factors affecting vaccination in pregnancy. Created by Claudio Rosa.

To optimize the protection offered to mothers and infants by maternal immunization, several factors that can affect this strategy must be better understood (Figure 2). The goal of this consensus paper written by experts in infectious diseases, vaccination and maternal immunization from different world regions is to summarize current evidence in the field of immunization during pregnancy and to highlight the knowledge gaps and prioritize future research strategies in order to optimize protection for the mother, fetus and the infant.

## Study Design

The main aim of this consensus paper is to discuss current knowledge regarding immunization during pregnancy and highlight the gaps that need to be addressed to ensure the highest protection for both the mother and their infants. References were identified through searches of PubMed for human studies published in English using the terms “immunization” or “vaccination” or “tetanus” or “tetanus disease” or “tetanus vaccine” or “pertussis” or “Tdap” or “pertussis immunization” or “pertussis vaccination” or “pertussis vaccine” or “Tdap vaccine” or “Tdap immunization” or “influenza” or “influenza vaccines” or “influenza immunization” or “maternal influenza vaccination” or “influenza vaccines in pregnancy” or “RSV” or “respiratory syncytial virus” or “GBS” or “GBS vaccine” or “Group B streptococcus” and “pregnancy.” Articles resulting from these searches and relevant references cited in those articles were reviewed. References were also provided by authors. Outcomes assessed were safety, immunogenicity, efficacy, and effectiveness of immunization during pregnancy against tetanus, pertussis, influenza, RSV, and GBS diseases. After the initial review, a meeting was held in Italy to discuss the current literature and knowledge gaps. A consensus on the content was reached after multiple rounds of revision among the authors.

## Ethics in Vaccine Trials During Pregnancy

Maternal immunization, and the use of medication in pregnancy in general, have been a focus of ethical deliberations for decades. Until recently, the ethical prevailing approach for immunization during pregnancy was based on the precautionary principle, which limits introduction of new intervention whose ultimate effects are uncertain. This precautionary principle-centered approach, combined with risk aversion among legal departments of vaccine manufacturers, led to exclusion of pregnant women from most vaccine trials for decades, leading to gaps in evidence of vaccine safety and efficacy among pregnant women. With an increasing focus on maternal immunization, there has been reconsideration of relevant ethical paradigms resulting in several recent developments in this area.

First, a report of the U.S. National Vaccine Advisory Committee's Working Group on Maternal Immunization recommended that “Relevant regulations, statutes, and policies…should be modified to indicate that pregnant women are not a vulnerable population for the purposes of ethical review” (13). This recommendation and concurrent policy action led to a change in the U.S. Code of Federal Regulations which had previously classified pregnant women as being inherently vulnerable to coercion (14). Second, recognizing that conventional paradigms often treated the risks and benefits of maternal immunization to mothers and infants as independent entities, a maternal interest-based paradigm was proposed by Chamberlain et al. (15). This paradigm recognizes the legitimacy of maternal interests in protecting their infants and the legitimacy of her taking measures that benefit only the fetus/newborn even if such measures do not have direct benefits for the mother herself. The third major development was creation of the Pregnancy Research Ethics for Vaccines, Epidemics, and New Technologies (PREVENT) working group. This multidisciplinary, international team of 17 experts developed a roadmap for inclusion of the interests of pregnant women in the development and deployment of vaccines (16). The underlying goal of these recommendations was to ensure that pregnant women's inclusion in vaccine trials is the default position and that any exclusions need to be justified rather than justifications being needed for inclusion of pregnant women.

Globally, a progress has also been made in the prioritization of immunization in pregnancy and the inclusion of pregnant women in vaccine trials. The WHO Strategic Advisory Group of Experts on Immunization (SAGE) recommended in 2012 that pregnant women should be highly prioritized for influenza vaccination in countries that consider initiating or expanding of seasonal influenza vaccine programs (17). In 2015, SAGE further emphasized the importance of the platform of immunization in pregnancy, as well the need to strengthen the delivery of vaccines administered during pregnancy (18).

These and other developments in ethical considerations for maternal immunization are likely to result in a more conducive environment for maternal immunization research and deployment. However, there are a few areas that require further deliberations (Table 1).

**Table 1:** Ethics areas related to immunization during pregnancy that require further deliberations.

Are there differential ethical considerations based on the gestational week of vaccination?
How is acceptable risk defined in pregnancy?
Can countries justify mass deployment of vaccines for use during pregnancy without an injury compensation program?

## Safety of Immunization During Pregnancy

Safety of vaccines administered during pregnancy needs to be evaluated for both the mother and her newborn, and is an important consideration for the mothers' willingness to receive a vaccine during pregnancy. There is a significant bulk of evidence to support the safety of immunization with tetanus toxoids (TT), the longest standing vaccine that is recommended during pregnancy. There is also an increasing body of evidence to support the safety of pertussis and influenza immunization during pregnancy (see below specific sections). However, continuous assessment and reporting of adverse events after immunization during pregnancy remains important, especially for relatively newly introduced maternal vaccines (e.g., pertussis), as it informs about rare events that might follow immunization. In addition, assessment of baseline pregnancy outcomes in unvaccinated women in different world regions and settings will help in establishing baselines to assess safety outcomes against.

Furthermore, there is significant heterogeneity and lack of consensus on adverse event reporting in maternal immunization studies. This is a challenge for comparing and pooling data from different studies. In an attempt to overcome this weakness, WHO and the Brighton Collaboration worked together to provide written guidance on how to conduct safety studies in the field of maternal immunization (19). The initiative termed the Global Alignment of Immunization Safety Assessment in Pregnancy (GAIA) project worked on standardizing the assessment of safety of vaccines in pregnancy with specific focus on LMICs (20). Specifically, this initiative proposed systematic data collection, specific case definitions of key obstetric and neonatal health outcomes, ontology of key terms and a map of pertinent disease codes. More recently, case definition and guidelines for data collection, analysis and presentation has been proposed for neonatal seizures, neurodevelopmental delay, chorioamnionitis and post-partum endometritis and infection by the GAIA and Brighton collaboration working groups (21–24). Future studies assessing safety of immunization during pregnancy should use the proposed terms and definitions. In addition, currently available data on safety of vaccination in pregnancy is derived from vaccines that were initially licensed in non-pregnant populations. Future vaccine trials will likely assess vaccines intended to be licensed primarily for use in pregnant women. This further emphasizes the need to standardize reporting of safety outcomes in maternal immunization trials. Thus, we recommend following the GAIA and Brighton collaboration guidelines for assessment and reporting of safety outcomes in maternal immunization trials.

## Key Factors That Influence Immunogenicity and Efficacy/Effectiveness of Immunization During Pregnancy

### Immune Responses of Pregnant Women to Vaccination

The immune system of a pregnant woman is adapted to allow for the survival of the semi-allogeneic fetus. Serum estradiol levels increase up to 500-fold during normal pregnancy (25), and the interplay between sex hormones and the maternal immune system in pregnancy is complex (Table 2). These changes might lead to the assumption that there are differences in immune responses to vaccines between healthy pregnant and non-pregnant women potentially leading to a lower immune response in pregnant women. However, studies comparing immunogenicity of vaccines in pregnant and non-pregnant women have generally not demonstrated decreased antibody responses in pregnant women. This has been the case for TT (40) and for the pertussis antigens in the combined tetanus, diphtheria, and acellular pertussis vaccine (Tdap) (41). However, results for influenza vaccines have been less consistent. Some studies carried out with influenza vaccines, including both the pandemic H1N1/2009 (pH1N1/2009) monovalent inactivated vaccine (MIV) and seasonal trivalent inactivated vaccine (TIV) preparations, show similar hemagglutination inhibition (HAI) seroconversion rates and antibody titers in pregnant and non-pregnant women (42–44). Other studies showed lower seroconversion rates and lower HAI geometric mean titers after vaccination of pregnant women when compared to non-pregnant women (45–47).

**Table 2:** Key changes in maternal adaptive immune system during pregnancy.

**Main changes**	**References**
Lower B cell levels in pregnant women compared with non-pregnant women	(26)
B cell lymphopenia in the third-trimester of pregnancy	(25)
Estrogen reduces B cell lymphopoiesis during pregnancy	(27, 28)
Decrease in B cell function	(29)
Decreased total IgG levels, especially during late pregnancy	(30, 31)
High estradiol levels promote T helper 2 cell responses	(32–34)
Elevated progesterone during pregnancy inhibits T helper 1 cell immune responses	(35)
A progressive shift from T helper 1 cell to T helper 2 cell responses	(36)
Decrease in T cell function	(37–39)

The effect of maternal immunization on cellular immunity has been less studied limiting conclusions. Proliferative and interferon-γ responses to the *Bordetella pertussis* (*B. pertussis*) antigens pertussis toxin (PT) 1 month after receipt of Tdap vaccine were not significantly different in pregnant and non-pregnant women and were comparable in both after 1 year (41). A small study showed that Natural killer cell and T-cell responses to inactivated influenza vaccination (IIV) were higher in a pregnant women compared to non-pregnant women (48).

### Trans-placental Transfer of Maternal Antibodies

Immunoglobulin G (IgG) is the dominant immunoglobulin isotype that crosses the placenta and contributes to maternally derived passive immunity during early infancy. In healthy pregnant women, IgG transfer across the placenta begins toward the end of the first trimester of pregnancy and increases as pregnancy progresses. IgG concentrations in the fetus are 5–10% of the maternal levels at 17–22 weeks gestation, 50% at weeks 28–32, and usually exceed maternal levels by 20–30% at term (49–52). The transplacental transfer of IgG is mediated by the neonatal Fc receptors (FcRn), localized in the syncytiotrophoblast that covers the villous tree of the placenta (53). FcRn regulates IgG transplacental transfer through binding to its constant domain and actively transport IgG into the fetal circulation. Several factors appear to affect the transfer of IgG across the placenta. IgG subclasses have differential efficiency of transfer across the placenta, defined as the antibody levels in the newborn divided by antibody levels in the mother. Based on studies from the 1990s, IgG1 is the subclass transferred with the highest efficiency, achieving higher levels in cord compared with the maternal blood, and this subclass is induced by vaccines containing protein antigens (54). IgG2 is transferred with the least efficiency, achieving lower cord than maternal blood levels, and is the dominant antibody induced by vaccines containing polysaccharide antigens (53, 55–58). Transfer of antibodies across the placenta can also be influenced by several clinical conditions in the mother and some of these health conditions are more prevalent in certain parts of the world. For instance, cord IgG levels were lower in infants from women with human immunodeficiency virus (HIV) infection (59), malaria infection (60), and hypergammaglobulinemia (61), compared with infants from women without those conditions, and these conditions are more prevalent in LMICs. In addition, the potential effect of toxoplasma and tuberculosis infection on the transfer of maternal antibodies has not been investigated. Furthermore, other maternal conditions, that have not yet been investigated, might also affect the structure of the placenta (e.g., gestational hypertension, gestational diabetes, smoking) and the transfer of maternal antibodies.

### Timing of Immunization

A number of factors should be considered when determining the ideal timing of vaccination in pregnancy including time-dependent safety when administered at different time points in gestation, time-dependent efficiency of transplacental transfer of vaccine-induced antibodies, interference with infants' immune response to vaccination and clinical efficacy/effectiveness (62, 63). Furthermore, the optimal timing of maternal immunization varies depending on who is the target for protection and when maximal protection is desired in the mother and/or the fetus/infant (62). For example, pregnancy is a well-known risk factor for severe influenza, being most severe during the third trimester of pregnancy (64, 65). Therefore, to maximize the protection for the mother, it is best to administer the influenza vaccine early in pregnancy and ideally prior to the peak of influenza seasonal activity. If the primary goal is to protect the infant, as for pertussis, the vaccine should be administered during a time period in gestation to provide optimal trans-placental transfer of antibodies, in order to ensure maximal protection against pertussis disease in early infancy (66). The risk for premature labor should also be considered as this population is at an increased risk for severe infections, such as pertussis and might not benefit from maternal vaccination if it happens late in gestation (67–69).

Based on the literature review and consultation among authors, a consensus on priorities for future research related to factors affecting immunization during pregnancy was reached (Table 3).

**Table 3:** Consensus on priorities for future research related to factors that influence the immunogenicity and efficacy/effectiveness of immunization during pregnancy.

**Immune responses of pregnant women to vaccination**
1. Immune response (quantity and quality of cellular and antibody immune responses) of pregnant women to vaccines with potential use in pregnancy in comparison with non-pregnant women
2. Immune response (quantity and quality of cellular and antibody immune responses) of pregnant women to vaccines with potential use in pregnancy at various stages of pregnancy in comparison with non-pregnant women
**Trans-placental transfer of maternal antibodies to fetus**
1. Create a better understanding of the molecular and cellular basis of maternal antibody transfer across the placenta, based on currently available vaccines for use in pregnancy, which would help the design of future vaccines that induce antibodies with optimal characteristics for transfer to the fetus
2. The induction of different vaccine-induced IgG subclasses should be evaluated early in the development of new vaccines designed for pregnant women
3. The effect of maternal health conditions on the transfer of vaccine-induced IgG subclasses should be assessed early in the development of new vaccines designed for pregnant women. This is especially important for some health conditions more prevalent in low-middle income countries such as poor nutrition, human immunodeficiency infection, malaria infection and hypergammaglobulinemia
**Timing of immunization during pregnancy**
1. The main target for protection in pregnancy (i.e., pregnant women and/or infant) and the time in gestation and/or infancy this maximal protection is desired have to be clearly defined for individual pathogens targeted for immunization
2. The safety of vaccination when administered in different stages during gestation
3. Time-dependent efficiency of transplacental transfer of vaccine-induced antibodies (quantity and quality)
4. Time-dependent clinical efficacy/effectiveness (for both term and preterm infants)

## Vaccines Currently Recommended for Pregnant Women

Vaccines currently recommended and used are aimed to protect against tetanus, pertussis and influenza diseases. Different vaccine formulations and dosages exist for use in pregnant women in selected countries in Europe, North America, South America, and Asia (Table 4).

**Table 4:** Formulations and dosages of common vaccines to protect against pertussis, and tetanus disease for use in pregnant women in selected countries in Europe, North America, South America, and Asia.

**Vaccine formulation**	**Antigen composition**	**References**	**Selected countries ^#^**
**Against tetanus**			
Td (MassBiologics)	Diphtheria toxoid: 2 Lf Tetanus toxoid: 2 Lf	(70)	South America: Honduras Asia: Thailand, Philippines, Malaysia Africa: Egypt, Gambia, Senegal, Gabon, Cameron, Botswana
TT adsorbed (Serum Institute of India)	TT ≥ 5 Lf	(71)	
**Against pertussis**			
Tdap (Adacel, Sanofi Pasteur)	Diphtheria toxoid: 2 Lf Tetanus toxoid: 5 Lf PT: 2.5 μg FHA: 5 μg PRN: 3 μg FIM: 5 μg	(70)	Europe: Belgium, Spain, United Kingdom, Italy North America: Canada, United States of America South America: Argentina, Brazil, Columbia, Chile, Mexico, Uruguay Asia: Singapore Africa: Australia and New Zealand
Tdap (Boostrix, GlaxoSmithKline)	Diphtheria toxoid: 2.5 Lf Tetanus toxoid: 5 Lf PT: 8 μg FHA: 8 μg PRN: 2.5 μg		
Against influenza^*^			
**Quadrivalent inactivated influenza vaccin**			Europe: Albania, Belgium, Hungary, Romania, Russian Federation, Spain, Sweden, United Kingdom, Italy North America: Canada, United States of America South America: Argentina, Brazil, Columbia, Ecuador, Bolivia, Mexico, Uruguay Australia and New Zealand Asia: Singapore, Thailand Africa: South Africa, Algeria
Afluria Quadrivalent (Seqirus) FluLaval Quadrivalent (GlaxoSmithKline) Flucelvax Quadrivalent (Seqirus) Fluzone Quadrivalent (Sanofi Pasteur)	Influenza A/Brisbane/02/2018 (H1N1)pdm09-like virus Influenza A/Kansas/14/2017 (H3N2)-like virus Influenza B/Colorado/06/2017-like (Victoria lineage) virus Influenza B/Phuket/3073/2013–like virus (Yamagata lineage) Dosage: Hemagglutinin 15 μG/dose (each virus)	(72)	
**Trivalent inactivated influenza vaccine**			
Fluad (Seqirus)	Influenza A/Brisbane/02/2018 (H1N1)pdm09-like virus Influenza A/Kansas/14/2017 (H3N2)-like virus Influenza B/Colorado/06/2017-like (Victoria lineage) virus Dosage: Hemagglutinin 15 μG/dose (each virus)	(72)	

*TT, Tetanus toxoid; Td, Tetanus diphtheria; Tdap, tetanus-diphtheria-acellular-pertussis; Lf, limit of flocculation; PT, pertussis toxin; FHA, filamentous hemagglutinin; PRN, pertactin; FIM, fimbria 2/3*.

* *Influenza vaccines compositions are reviewed each year and updated as needed. Composition presented is for 2019-20 influenza season*.

# *Source: World Health Organization website: https://apps.who.int/immunization_monitoring/globalsummary*.

### Vaccines Against Tetanus

The World Health Organization (WHO) recommends that if a pregnant woman has never received a tetanus-toxoid -containing vaccine (TT-CV) (e.g., Diphtheria-Tetanus-Pertussis [DTP], Diphtheria-Tetanus [DT], Tetanus-diphtheria [Td], TT) or her vaccination status is unknown, she should receive two TT (or Td) vaccine doses 4 weeks apart during pregnancy, with the second dose given at least 2 weeks before delivery. Based on WHO recommendations, five total doses are likely needed for protection throughout the childbearing years so a third dose is given 6 months after the second dose, and two additional doses are recommended to be given during the next 2 years or during two subsequent pregnancies (73). For women who have received 1–4 TT-CV doses prior to their pregnancy, one TT-CV dose is recommended during each subsequent pregnancy to a total of five doses. However, this vaccination schedule and policy has never been formally evaluated in clinical trials.

#### Safety

Several studies have demonstrated TT-CVs to be safe in pregnancy (74–76). As the current pertussis-containing vaccines administered in pregnancy are part of multicomponent formulations that include TT, safety assessments of pertussis-containing vaccines in pregnancy also provide information on the safety of the TT component (see below discussion under pertussis vaccines) (77). Safety was demonstrated even when the most recent TT-CV was administered within 2 years prior to vaccination in pregnancy (76).

#### Immunogenicity

Several studies have shown that following maternal immunization with TT-CVs, anti-TT IgG is actively transferred across the placenta, leading to protective levels in the infant (77–80). Vaccination with TT induces IgG1 (54, 81), which are efficiently transferred across the placenta. Approximately 80% of maternal antibodies remain present in infants 1 month after delivery; thus, protection is maintained until a primary vaccination course is commenced and is maximal during the most vulnerable period when umbilical infections may occur (82).

If a Tdap vaccine in pregnancy is being considered to replace a single dose of TT vaccine in some settings, in order to provide dual coverage for pertussis and tetanus disease, it is important to assess the immunogenicity of Tdap in inducing anti-TT IgG compared with TT or Td formulations. In a small study from Vietnam, vaccination with Tdap in pregnancy resulted in higher cord anti-TT IgG levels compared with vaccination with TT, however, this difference did not persist at 2 months of age (83). These results are reassuring that replacing TT with Tdap is not expected to result in inferior immunogenicity against tetanus.

#### Effectiveness

Both maternal and neonatal tetanus were very common in most developing countries even into the 1980's. In 1989, the WHO called for the elimination of maternal and neonatal tetanus by the end of the century. At that time, 59 countries reported maternal and neonatal tetanus. As part of the MNTE program, and along with safer birth techniques and effective immunization strategies in children and adults, more than 150 million women were vaccinated against tetanus during pregnancy. Altogether, these practices contributed to the elimination of maternal and neonatal tetanus in 45/59 countries as of the end of 2018 (84, 85). However, 14 countries, mainly in Africa, still have residual maternal and neonatal tetanus, highlighting that additional efforts are required to extend maternal immunization, immunization of children and adolescents, and other hygienic measures aimed at improved cord-care. The WHOs most updated goal is to achieve maternal and neonatal tetanus elimination by 2020 which will be difficult to achieve (85).

Based on the literature review and consultation among authors, a consensus on priorities for future research related to immunization against tetanus during pregnancy was reached (Table 5).

**Table 5:** Consensus on priorities for future research related to vaccination against tetanus during pregnancy.

**Vaccines against tetanus disease**
**Immunogenicity**
1. The immunogenicity of different Tdap formulations in pregnancy compared with TT and Td in countries where TT/Td immunization is given in pregnancy and Tdap immunization in pregnancy is being considered
2. Immunogenicity of different dosing regimens (number of doses) of tetanus vaccination during pregnancy, especially in settings where vaccination against tetanus in childhood is high

*TT, Tetanus toxoid; Td, Tetanus diphtheria; Tdap, tetanus-diphtheria-acellular-pertussis*.

### Vaccines Against Pertussis

#### Safety

Data on tolerability and safety of pertussis immunization during pregnancy are reassuring (86). This has been demonstrated with different Tdap vaccine formulations regardless of the number of pertussis antigens included in the vaccines (77, 87–96). Specifically, no increased risk for the development of severe maternal adverse events (e.g., postpartum endometritis, preterm delivery, and preterm premature rupture of membranes) or fetal and neonatal outcomes (e.g., low birth weight, very low birth weight, small for gestational age, birth defects, and need for neonatal intensive care unit admission) has been reported. However, a small increased risk of chorioamnionitis among Tdap-vaccinated women (relative risk [RR] 1.19, 95% CI, 1.13 to 1.26) was documented in one study (89). In another study using the Vaccine Adverse Event Reporting System database, the majority of these women with chorioamnionitis had at least one risk factor for this complication (97). In addition, there was limited supportive evidence for a chorioamnionitis diagnosis on chart review and the risk of preterm birth (a concern after chorioamnionitis) was not higher among Tdap recipients. Therefore, the association between this complication and vaccination during pregnancy has been debated. However, a recent study reported a small increase of chorioamnionitis in pregnant women who received Tdap vaccine during pregnancy with a RR of 1.11 (95% CI: 1.07–1.15); but the absolute risk was still quite low, at 2.8% (98). Ongoing studies are currently evaluating the potential association between Tdap vaccination during pregnancy and chorioamnionitis. In view of the recommendation to vaccinate against pertussis during each pregnancy, it has been shown that repeated Tdap vaccinations in consecutive pregnancies are well-tolerated (76).

#### Immunogenicity

Vaccination against pertussis in pregnancy has been achieved using Tdap formulations that include mostly three or five *B. pertussis* antigens. Antibodies against all *B. pertussis* antigens included in the Tdap vaccine have been shown to reach peak levels at the end of the second week after Tdap administration in non-pregnant women of childbearing age, and this peak is followed by a rapid decline (99). In pregnant women, studies have shown a significant increase in *B. pertussis-*specific antibody levels 1 month after Tdap vaccination, also with a significant decline, within the first year after maternal vaccination (41, 100, 101). Thus, the persistence of antibodies after a single dose of Tdap vaccine in pregnancy is short and does not probably ensure infant protection during consecutive pregnancies. Therefore, vaccination is currently recommended in every pregnancy.

Vaccination with *B. pertussis* antigens induces mainly IgG1 antibodies (102, 103) which are actively across the placenta to the newborn resulting in higher antibody levels in the term newborn than in the mother (77, 80, 104).

Pertussis toxin is a major virulence factor of *B. pertussis* and is potentially responsible for both local and systemic responses (105). Administration of humanized neutralizing anti-PT monoclonal antibodies have been shown to abolish disease manifestations in mice and non-human primates (106). Maternal immunization with a monocomponent PT vaccine protected newborn baboons against pertussis following respiratory challenge with *B. pertussis* (107). In human, low anti-PT IgG levels have been associated with high susceptibility to pertussis (108). However, antibody levels that confer protection against human pertussis disease have not been defined. In addition, the number and type of *B. pertussis* antigens are required for pregnant women in order to provide clinical protection to the infant has not been clearly established.

#### Timing

A study conducted in Thailand showed that vaccination earlier in pregnancy was associated with higher *B. pertussis*-specific cord antibody levels (109). Furthermore, three other studies found that vaccination during the early third trimester of pregnancy is associated with higher cord anti-*B. pertussis*-specific IgG levels than immunization during late third trimester (110–112). In addition, one study showed that anti-PT and anti-FHA IgG levels were higher in cord blood of mothers vaccinated between 13 and 25 weeks gestation compared to those immunized after 25 weeks gestation (113). This was also observed in preterm infants (114). In addition, avidity of cord anti-PT IgG was higher when mothers were vaccinated in the early third trimester compared with late third trimester (115, 116), although this finding was not observed in a third study (117). Therefore, more data are needed to address this controversy, including also data from vaccination at earlier time points in pregnancy. Moreover, because the role of antibody levels and avidity in protection against pertussis is not conclusive to date, interpretation of the above studies requires caution.

#### Effectiveness

Effectiveness of maternal immunization for prevention of pertussis in young infants has been well-studied. In England, vaccine effectiveness was 91% in the reduction of laboratory-confirmed cases in infants <3 months of age (6), and 93% in prevention of laboratory-confirmed cases in infants <8 weeks of age (118). In the US, effectiveness among infants <8 weeks of life ranged between 85 and 91% (7, 119, 120). In addition, disease was significantly less severe among infants from vaccinated mothers (119). In Spain, a case-control study reported VE to be 90% against laboratory-confirmed pertussis infection in infants <3 months of age (121), while in Australia it was 69% in infants <3 month of age (122). In Brazil, vaccine effectiveness was reported to be 82.6% for the prevention of clinical pertussis in infants <2 months of age, confirming the success of the maternal pertussis immunization strategy also in middle-income countries (123).

Based on the literature review and consultation among authors, a consensus on priorities for future research related to immunization against pertussis during pregnancy was reached (Table 6).

**Table 6:** Consensus on priorities for future research related to vaccination against pertussis disease during pregnancy.

**Safety**
1. The association between receipt of Tdap in pregnancy and chorioamnionitis
**Immunogenicity**
1. Assessment of immune correlates for protection against pertussis disease (e.g., *Bordetella pertussis* –specific antibody levels)
2. *Bordetella pertussis* antigens to be included in pertussis vaccines for maternal immunization to provide sufficient clinical protection to the infant
3. The need for immunization against pertussis disease in subsequent (3rd or more) pregnancies.
4. Comparative studies comparing different pertussis vaccine formulations (e.g., Tdap vs. aP stand-alone vaccines)
5. Role of previous vaccination of the mother with whole cell or aP vaccines in the immune response to maternal pertussis vaccination
**Timing**
1. The effect of timing of vaccination on the function of anti-B*ordetella pertussis* antibodies transferred to infants
2. The immunogenicity of stand-alone aP given in different times in pregnancy
**Effectiveness**
1. Burden of pertussis disease in infancy in low and middle-income countries
2. The effectiveness of maternal immunization program in low and middle-income countries if pertussis immunization in pregnancy is implemented
3. Assess the eventual role of previous vaccination with whole cell or acellular pertussis to the mother on vaccine effectiveness
4. Vaccine effectiveness of various Tdap formulations

*Tdap, tetanus-diphtheria-acellular-pertussis; aP, acellular pertussis*.

### Vaccines Against Influenza

#### Safety

There is an extensive body of evidence in the literature from both HICs and LMICs that confirm the safety of maternal influenza vaccination (124–129) [reviewed in (130)]. During the H1N1 influenza pandemic, data from Sweden and Argentina found that both AS03-adjuvanted and MF59-adjuvanted -H1N1 influenza vaccines were not associated with increased risk for low-birth weight or preterm birth or low Apgar score (131, 132). A meta-analysis including studies using both adjuvanted and non-adjuvanted influenza vaccines found lower estimates of still birth after maternal influenza vaccination and no association with an increased risk of spontaneous abortion (133). However, a small case-control study in the US over two influenza seasons (2010–11, 2011–12) found an increased risk of early spontaneous abortion in a group of women who had received influenza vaccination in the first trimester of pregnancy, although cases had other risk factors for spontaneous abortion (older age, pervious history of spontaneous abortion, smoking); thus the causal relationship between influenza vaccination and this complication has been questioned (134). To further support the safety of influenza vaccination in pregnancy, three Bill & Melinda Gates Foundation funded studies from South Africa (135), Mali (136), and Nepal (137), and recent studies and systematic reviews found that maternal influenza vaccination was not associated with an increased risk of fetal death, spontaneous abortion, or congenital malformations (138–141).

Furthermore, concomitant or sequential vaccination with Tdap and influenza vaccines has also been shown to be safe and not associated with differences in medically attended acute events in pregnant women or adverse birth outcomes (142).

#### Immunogenicity

Influenza vaccination preferentially induces IgG1 subclass antibodies (143), and studies have shown increased levels of influenza-specific hemagglutinin antibodies in neonates born to women given a monovalent (pH1N1/09) or seasonal TIV during pregnancy, suggesting efficient transplacental transfer of influenza-specific antibodies (144–146). Importantly, seroconversion rates were lower after administration of TIV in women living with HIV than in women without HIV, and hemagglutination-inhibiting antibodies (HIA) titers were lower in HIV-exposed infants (146).

The kinetics of influenza antibody decline in the infant vary according to the influenza virus and the levels of transferred antibodies, and thus the duration of protection is not precisely defined. Some data indicate that maternally-derived HIA against seasonal influenza viruses have a half-life of approximately 45 days in infants after maternal vaccination and that these antibodies decline to levels similar to those detected in infants born to unvaccinated women by 16 weeks of age (147, 148). This is consistent with higher protection from laboratory-confirmed influenza disease among infants of vaccinated mothers during the first 2–3 months of age (135, 136).

In another study, children born to mothers vaccinated with an adjuvanted pH1N1 vaccine had antibody levels that remained elevated above the correlate of protection for adults (HIA titer > 1:40) up to 5 months (149). However, the interpretation of influenza immunogenicity studies are complicated as the correlate of protection against infection in infants has not yet been established and is likely to be different and higher than the correlate of protection used for adults (150). This is an area of controversy, where more research is needed to define the correlate(s) of protection against influenza disease in infants, which is important as currently available pediatric influenza vaccines are recommended in certain settings from 6 months of age onwards.

#### Timing

The optimal timing for maternal influenza immunization has not been established, and recommendations (e.g., CDC, ECDC, WHO) allow administration at any time during pregnancy (17, 151, 152). Importantly, since influenza is a seasonal disease (except in tropical regions, where influenza disease may occur throughout the year) and the goal of vaccination also serves to protect the mother, the actual determination of timing may depend on factors other than optimizing antibody transfer to the infant. Jackson et al. reported lower antibody levels at birth in infants of mothers vaccinated earlier during pregnancy (144). On the other hand, Sperling et al. did not find a significant association between the gestational age at vaccination and the seroconversion rates following influenza vaccination in pregnant women. However, maternal seroconversion rates were slightly lower in women immunized in the first trimester than in those given the vaccine in the late third trimester (153). In another study, a higher level of transplacental transfer of antibodies was associated with a longer interval between vaccination and delivery in pregnant women vaccinated against influenza after 20 weeks gestation (146). Blanchard-Rohner et al. showed that receipt of influenza vaccine at least 2 weeks before delivery increased umbilical cord HIA titers and seroprotection rates in newborns (154). Finally, Katz et al. found no significant differences in influenza HIA titers in cord sera of women vaccinated early (17–25 weeks gestation) or later (26–34 weeks gestation) in randomized trials during pregnancy (155).

#### Efficacy

Influenza can be a severe disease for pregnant women, neonates and young infants. The severity of infection increases as pregnancy advances, with the greatest maternal risk occurring during the third trimester of pregnancy (156, 157). Young infants on the other hand, have been shown to experience the highest rates of influenza-related hospitalization (158) and death (159) among children with influenza infection.

Multiple studies have shown that administration of an IIV during pregnancy reduces the risk of influenza in pregnant woman by ~35–50% (135, 160–162). The efficacy of maternal influenza vaccination against laboratory-confirmed influenza in infants below 6 months of age also varies in different trials conducted at different geographic sites. Efficacy has been 63% (95% CI, 5–85) in Bangladesh (162), 49% (95% CI, 12–70) in South Africa (135), 33% (95% CI, 4–54) in Mali (136), and 30% (95% CI, 5–48) in Nepal (137). Efficacy against laboratory-confirmed influenza in infant was higher in the first 2–3 months of life and in the range of 70–80% in 2 RCTs from South Africa and Mali (135, 136). Observational studies carried out in the USA (163, 164) and England (165), reported reductions of laboratory-confirmed influenza in children born to vaccinated mothers ranged from 41 to 71%. A recent meta-analysis reported that maternal influenza vaccination reduced the risk of laboratory-confirmed influenza infection in infants by 48% (95% CI, 33–59) (166). In addition, maternal influenza vaccination was associated with a reduction in all-cause severe pneumonia in infants. An analysis of three Bill & Melinda Gates foundation -funded clinical trials conducted in Nepal, Mali and South Africa including 10,002 mothers and 9801 live-born eligible infants concluded that the pooled incidence rate of severe pneumonia up to 6 months of age was 20% lower in infants born to women vaccinated with IIV compared with infants born to women unvaccinated in pregnancy (incidence rate ratio [IRR]: 0.80; 95% CI: 0.66–0.99) (167). However, it should be noted that few of these cases had influenza identified despite testing suggesting that influenza vaccination during pregnancy might have benefits beyond the prevention of classical influenza disease.

The efficacy of IIV in pregnancy in the prevention of maternal and infant influenza disease varies depending on the setting as well as the match of the vaccine utilized to circulating influenza strains. The majority of efficacy data are derived from studies performed in LMICs when compared to HICs. While influenza disease is seasonal in countries with temperate climates (e.g., Europe, North America), there is no seasonal pattern in tropical countries.

Altogether, current data on safety, immunogenicity, and efficacy of maternal IIV vaccination, for the pregnant women and their infants has resulted in pregnancy as a potential indication in the vaccine label by the European Medicines Agency as of July, 2019 (168). In Australia, categorization of influenza vaccines given during pregnancy has changed to category A (no proven harmful effects) (169). Other individual countries will have their own considerations.

Based on the literature review and consultation among authors, a consensus on priorities for future research realted to immunization against influenza during pregnancy was reached (Table 7).

**Table 7:** Consensus on priorities for future research related to vaccination against influenza disease during pregnancy.

**Immunogenicity**
1. Correlate(s) of protection against influenza disease in infants
2. The duration of protection conferred by vaccination in pregnancy in infants. This needs to take into account seasonality in different settings (tropical regions vs. temperate climate regions)
3. Evaluation of more immunogenic influenza vaccines in pregnant women to optimize antibody transfer to their infants
**Efficacy/Effectiveness**
1. The development of more immunogenic influenza vaccines to optimize protection of young infants
2. Evaluate vaccine-efficacy against non-specific (all-cause) lower-respiratory tract infections

## Impact of Maternal Immunization on Infants' Immune Responses to Immunization

High levels of vaccine-induced maternally-derived antibodies have the potential to reduce the infants' humoral immune responses by inhibiting antibody generation after the infant's own vaccination, leading to lower antibody levels/titers later on in the infant (170, 171). This phenomenon is called “interference” or “blunting” and has been described for the same vaccine antigens used by mother and infant, as well as for conjugated vaccines administered in infancy (172). Data from the 1990s showed that the administration of *Haemophilus influenzae* type b (Hib) polysaccharide or Hib conjugated vaccines in pregnant women was associated with mild inhibition of infants' immune responses to Hib conjugated vaccines (173). Differences in antibody responses in infants born to vaccinated compared with unvaccinated mothers were minimized following the booster dose. An analysis of the genetic repertoire of the light chain of antibodies to the polysaccharide vaccine demonstrated no differences between infants born to immunized women compared with non-immunized women (174). There was no evidence of inhibition of “priming” of the infants' immune system to Hib in these studies.

### Tetanus-Containing Vaccines

Most data on the impact on TT-CVs in infancy are derived from studies that used Tdap formulations in pregnancy and measured anti-TT IgG levels after infant vaccination. These studies found inconsistent results. Some showed significantly lower anti-TT levels after primary immunization in infants born to Tdap-vaccinated women compared to infants from unvaccinated women whilst other studies showed equal or even significantly higher anti-TT levels in infants born to Tdap-vaccinated women compared to infants from unvaccinated women (77, 80, 175–177). However, this inhibition found in some studies did not result in a reduction of the percentage of infants with seroprotective anti-TT antibody levels.

The effect of different TT-CV formulations used in pregnancy (Tdap vs. TT/Td) on immune responses to tetanus-containing vaccines in infancy is of importance in countries where a replacement of the existing tetanus vaccination program by a Tdap vaccination program is being considered. A small study in Vietnam reported higher anti-TT levels after primary immunization with tetanus-containing vaccines in infants born to Tdap-vaccinated pregnant women compared to infants born to TT-vaccinated pregnant women (83). A study from Canada found no difference in anti-TT levels after primary and booster immunization in infants born to Tdap-vaccinated pregnant women when compared to Td-vaccinated pregnant women (104). These data suggest that Tdap, when compared to TT or Td in pregnancy, is not associated with lower anti-TT IgG levels after primary and booster immunization with tetanus-containing vaccines in infancy. However, in order to provide a definite conclusion, formal studies should be conducted with the aim to address this question as the primary outcome.

Several vaccines are conjugated to TT as a carrier protein (e.g., Hib vaccines, meningococcal vaccines) and thus vaccine-induced immune responses to these vaccines in infant born to Tdap-vaccinated pregnant women might also be affected. Hib anti-polyribosylribitol phosphate (PRP) levels were higher after primary immunization with Hib TT-conjugated vaccine in infants born to Tdap-vaccinated pregnant women when compared to infants of unvaccinated mothers (175, 178).

One study found no differences between anti-Men C antibody levels after primary immunization with meningococcal C TT-conjugated vaccine in infant born to Tdap-vaccinated when compared to unvaccinated pregnant women (178). More studies are needed to investigate the potential effect of tetanus-containing vaccines administered in pregnancy on infants' immune response to vaccines conjugated to TT.

### Pertussis Vaccines

Studies have shown that Tdap immunization in pregnancy is associated with decreases in humoral immune responses to infants' immunization with acellular pertussis (aP) containing vaccines. Several studies describe significantly lower anti-PT IgG levels in infants born to Tdap-vaccinated pregnant women after the completion of primary immunization, while results were less consistent after booster immunization (77, 80, 83, 104, 175–177). Results from these studies showed also interference to other pertussis antigens (FHA, pertactin, fimbria 2/3) after primary immunization while results were inconsistent after booster immunization.

Most studies investigating the potential modification of infants' immune responses to aP vaccines have been performed in HICs, with the exception of one study from Vietnam (83, 177). It is important to note that the degree of reduction in immune responses to wP infant vaccines might be different than to immunization with aP infant vaccines. The use of wP vaccines but not aP vaccines was associated with a substantial reduction in the subsequent infant antibody response to PT in infants born to mothers with high levels of maternally-derived anti-PT antibodies (179). In another study, there was no correlation between low anti- *B. pertussis* antibody levels at delivery in infants born to unvaccinated women and their anti-*B. pertussis* antibody levels after wP vaccination (180).

A recent study reported that Thai infants born to unvaccinated mothers and subsequently vaccinated with wP vaccines, had higher anti-*B. pertussis*-specific antibody levels after primary and booster vaccination than infants born to women vaccinated with Tdap during pregnancy and vaccinated with wP vaccines (181). In addition, infants born to women vaccinated with Tdap in pregnancy and vaccinated with wP vaccines had lower anti- *B. pertussis*-specific antibody levels after vaccination when compared with infants born to vaccinated mothers and vaccinated with aP vaccines (181).

Altogether, these results indicate that infants born to Tdap-vaccinated mothers might be at increased risk for pertussis later in life. However, surveillance data from the US and UK did not demonstrate any increase in the number of pertussis cases later in infancy after the introduction of the maternal immunization program suggesting a possible lack of clinical significance of this interference (6). Interpretation of interference to wP immunization is more challenging in LMICS compared with HICs due to the lack of comprehensive surveillance systems in some countries (182).

Because vaccines against pertussis that are currently used in pregnancy also contain dT, interference might also be extended to diphtheria-containing vaccines administered in infancy. Data on this respect have been inconsistent, with some studies reporting significantly lower anti-diphtheria toxin antibody levels in infants born to Tdap-vaccinated women when compared to infants born to unvaccinated women, while other studies did not report this effect (77, 80, 83, 104, 175–177). It is also important to note that Tdap immunization in pregnancy, likely due to anti-DT antibodies, is associated with lower anti-pneumococcal capsular polysaccharide levels after immunization with pneumococcal vaccines (PCVs) conjugated to a non-toxic diphtheria toxin mutant (CRM197), although, this did not result in lower seropotection levels for most serotypes (175, 183). Surveillance will be key to assess whether this interference has any impact on pneumococcal disease burden.

If long-term surveillance data would indicate that interference is clinically significant, strategies to mitigate the effect of interference will need to be evaluated. Timing of vaccination in pregnancy is an important modifiable variable and should be investigated. Delaying primary infant vaccination is another approach and has been recently implemented in The Netherlands in infants born to Tdap-vaccinated mothers. In addition, stand-alone pertussis vaccines (without TT, dT) should be investigated in clinical trials (184) as these vaccines might lessen the concern of interference to TT and DT components and vaccines conjugated to those proteins as carrier proteins.

### Influenza Vaccines

Data on the potential impact of maternal influenza immunization on the immune response of infants to their immunization against influenza are scarce as influenza vaccines are administered in infants older than 6 months, when most maternally-derived antibodies already have waned from infant's circulation. Earlier studies performed to assess immunogenicity of influenza vaccination in infants younger than 6 months old found that post vaccination seroprotection rates (titer ≥ 1:40) were higher in infants who received IIV at 6 months of age when compared to infants who received vaccination during 6–12 weeks of age (185). Another prospective, open-label study in which 2 doses of a TIV were administered to healthy infants aged 3–5 months found a 4-fold increase in antibody titers to be significantly more common in children who were seronegative (pre-vaccination titers <1:8) at enrollment than in those with pre-vaccination titers ≥1:8 (186).

### Mechanism of Interference

Mechanism of interference between maternally-derived antibodies and infant's immune responses to subsequent immunizations has not been fully explored (187). Some proposed mechanisms include inhibition of B cell response to vaccine antigens through epitope masking by maternal antibodies (172) and neutralization of vaccine antigens (187, 188). Inhibition of B cell activation through crosslinking of FcγRIIB to the B-cell receptor on B cells has also been proposed. Specifically, vaccine antigen–antibody complexes cross-link the B-cell receptor (which recognizes the variable region of the antibody) with the Fcγ receptor IIB (which recognizes the constant region of the antibody), thus inhibiting antigen specific B-cell activation (189). Furthermore, vaccine antigen-antibody complexes removal by macrophages has been suggested although no evidence has been provided to support this hypothesis. Using influenza vaccination in pregnancy as a model, it was recently shown in mice that maternal antibodies do not prevent activation of B cells or the formation of the germinal center. However, maternal antibodies reduced the number of B cells that differentiate to plasma cells and memory B cells (190). Whether these results apply to human infants and other antigens needs to be determined. Finally, while B cell responses are inhibited in the presence of maternal antibodies, scarce data support that T cell responses are detected in the presence of maternal antibodies (191).

## Impact of Maternal Immunization on the Neonatal Immune System

The impact of maternal vaccination on the fetal/neonatal immune system, beyond the trans-placental transfer of IgG, has not been well-studied. *In utero* priming of the fetal immune system after vaccination against influenza in pregnancy has been reported. IgM antibodies against influenza vaccine antigens were detected in nearly 40% of cord blood specimens of newborns born to women vaccinated with IIV in pregnancy (192). As IgM antibodies do not cross the placenta, these antibodies are of fetal origin. In addition, using MHC tetramers, HA-specific CD4^+^ T cells were also detected in cord blood, further supporting the “*in utero* priming hypothesis” after maternal immunization (192). Additional studies are needed to further assess the possibility of priming of fetal immune system to *B. pertussis* antigens after immunization in pregnancy.

Based on the literature review and consultation among authors, a consensus on priorities for future research related to the effect of immunization during pregnancy on infants' immune responses was reached (Table 8).

**Table 8:** Consensus on priorities for future research related to the impact of maternal immunization on *in-utero* immune system and infants' immune responses to immunization.

**Infants' immune responses to TT-containing vaccines**
1. The impact of anti-TT maternally-derived antibodies on infants' responses to tetanus-containing vaccines administered during infancy and whether this is affected by vaccine formulation given to pregnant women (TT vs. Td vs. Tdap)
**Infants' immune responses to DT-containing vaccines**
1. The impact of anti-DT maternally-derived antibodies on infants' responses to vaccines conjugated to DT mutants as a carrier protein (e.g., CRM197-conjugated vaccines) administered during infancy
**Infants' immune responses to pertussis vaccines**
1. Clinical significance of interference to pertussis immunization in pregnancy
2. If interference is found to be clinically significant, modifiable factors that can mitigate interference need to be explored
3. The effect of timing of vaccination during pregnancy on interference
4. The impact of a stand-alone pertussis vaccine (no TT, DT) on infants' immune responses to pertussis vaccine administered during pregnancy
**General**
1. The mechanism of inhibition of maternally-derived antibodies on infants immune responses to their vaccination
2. The effect of maternally derived antibodies on infant T cell responses
3. The potential priming of the fetal immune system to vaccine antigens after immunization during pregnancy and its effect on training neonatal immune system

*TT, Tetanus toxoid; Td, Tetanus diphtheria; Tdap, tetanus-diphtheria-acellular-pertussis; DT, Diphtheria toxoid*.

## Future Vaccines for Immunization During Pregnancy

In addition to tetanus containing, pertussis containing and influenza vaccines currently used in pregnancy, multiple novel GBS and RSV candidate vaccines are under development for use in pregnant women (193). Infection with other pathogens (e.g., dengue virus, Zika virus) during pregnancy is associated with a significant risk of adverse fetal outcome (194–196), and thus vaccines developed with the goal to prevent these congenital infections might prove to be an important preventative strategy. However, these not part of this consensus paper and are reviewed elsewhere (197, 198).

### Group B *Streptococcus* Vaccines

GBS colonization in pregnant women is associated with an increased risk of premature birth, birth asphyxia, stillbirths, and invasive GBS disease in newborns during the first week of life (early-onset disease, EOD). Newborns of mothers colonized with GBS are at higher risk of developing meningitis and sepsis (199). Although intrapartum antibiotic prophylaxis is effective in preventing GBS EOD, it is not effective in preventing late onset disease (LOD, >7–90 days of age) and it might be associated with dysregulation of the infants' gastro-intestinal microbiome (200). Importantly, identification and treatment of colonized mothers can be difficult and expensive, particularly in LMICs, where the incidence of neonatal invasive GBS disease is higher compared to HICs (201). Development of GBS vaccines for immunization in pregnancy and its use in LMICs has been identified as a priority by the WHO (202).

Vaccines based on the capsular polysaccharide of the most common GBS strains conjugated to a carrier protein (e.g., TT or a non-toxic mutant of diphtheria toxin) are the most studied candidate vaccines (203). A recent systematic review of clinical trials evaluating candidate GBS vaccines concluded that these candidate GBS vaccines are safe and well-tolerated in pregnant women and non-pregnant adults, may reduce vaginal colonization and induce antibody titers against the GBS strains included in the vaccine at a significantly higher level than that detected in unvaccinated controls (203). Moreover, antibodies induced by GBS vaccines showed high longevity and were able to promote GBS opsonophagocytosis *in vitro* (203).

Several challenges for the development of GBS vaccines for maternal immunization remain unsolved. There are only 10 known GBS serotypes, of which 6 are associated with 98% of all described strains that cause invasive disease and even a trivalent vaccine (Ia, Ib, and III) would provide coverage for 80% of all global invasive disease cases (204). The prevalence of different GBS serotypes may vary in different countries, however, the most common serotypes (Ia, Ib, II, III, IV, and V) are dominant globally, with only Asia reporting a slightly higher proportion of cases due to one additional serotype (VII) (205). The distribution of serotypes responsible for early and late -onset GBS disease also varies, with the most common serotypes being III and Ia (206). Correlates of protection for the different GBS serotypes against the various clinical conditions associated with the pathogen (i.e., colonization, maternal and infant disease) are not precisely defined (207), and these correlates may vary by serotype (207). Furthermore, transplacental transfer of antibodies might be affected by the characteristics of the vaccine (conjugated vs. unconjugated), the carrier protein used for conjugation, and the presence of underlying diseases in the mother which can reduce transfer, such as HIV infection (208).

Phase 1b/2 clinical trials have shown that vaccination of pregnant women with a trivalent GBS vaccine (serotype III, Ia, and Ib conjugated to CRM197) induces anti-GBS antibodies that are transferred to the newborn at delivery (208–210). Other phase 1/2 clinical trials are currently evaluating multi-serotype vaccines, including a hexavalent vaccine (serotypes Ia, Ib, II, III, IV, V) that cover 98% of strains associated with invasive GBS disease in both a non-pregnant population (NCT03170609) and in pregnant women (NCT03765073).

Finally, the clinical effectiveness of GBS vaccines in pregnant women and neonates has not been determined. Considering the relatively low incidence of invasive GBS disease, especially in HICs, the pathway of licensure of a GBS vaccine targeted at pregnant women with the main objective of protection of their infants against early and late-onset invasive GBS disease is likely to require an alternate approach than conventional efficacy trials. This would include demonstrating the safety of the vaccine in pregnant women (~3,000–4,500 participants), and benchmarking their immune responses to a serological endpoint associated with reduced risk for invasive GBS disease. Studies are currently underway in LMICs and HICs, which are investigating the association of maternal-derived serotype-specific IgG (using a standardized assay) and threshold associated with 80–90% risk reduction for invasive GBS disease.

As current GBS vaccines that are under development are conjugated to TT or the DT mutant CRM197, it will be important to investigate whether these vaccines given to pregnant women may result in interference to infant vaccines conjugated to these carrier proteins and given in infancy (e.g., PCV, Hib, and Meningococcal vaccines). Current evidence suggests that CRM197-conjugated GBS vaccine administered in pregnancy did not affect infants' immune responses to PCVs (211).

### Respiratory Syncytial Virus

RSV is the most common cause of severe lower respiratory tract infections (LRTIs) in young children worldwide with a disproportionate high burden of disease in LMICs (e.g., higher case-fatality rate) (212). Preterm infants and infants with underlying severe chronic heart or lung disease are at higher risk of severe RSV infection, leading to hospitalization and death. A monoclonal antibody directed against the RSV fusion (F) protein has been administered to high-risk populations to prevent RSV-related morbidity in infants in high-income countries (213, 214). However, this strategy is highly expensive and its effectiveness varies ranging between 48 and 96% in the prevention of RSV-related hospitalization in high-risk children (215, 216). In addition, overall more healthy children are infected with RSV each year than high-risk children. A novel prolonged half-life anti-RSV monoclonal antibody may prove to be more effective in preventing RSV disease in infancy (217).

Recently, several new vaccines, including live-attenuated, gene-based vector vaccines, and particle-based vaccines, have been developed and found to be safe and well-tolerated in the non-pregnant population (11, 193). Hence, as most of the cases of severe RSV infection occur in the first 3 months of life, it is unlikely that infants' immunization can provide sufficient and timely protection. Therefore, maternal immunization is considered as a suitable strategy for prevention of RSV disease in young infants (11, 218).

Studies on RSV-F protein in pregnant women have shown that these vaccines are safe and immunogenic in pregnant women (219, 220). The use of these RSV vaccines in healthy pregnant women is further supported by evidence that maternal RSV neutralizing antibodies are efficiently transferred from the mother to the newborn, with levels at delivery that are similar or higher in the cord blood compared with the maternal blood at delivery (219, 220). However, the association between higher cord RSV neutralizing antibody levels and the reduction of risk for RSV LRTI in the infant is not clear, and no definitive correlates of protection have been defined so far (221–223). Vaccines containing the RSV-F protein in pregnant women have shown that these vaccines are safe and immunogenic in pregnant women (219, 220).

A phase 3, randomized, placebo controlled trial including 4,636 pregnant women has been conducted in 11 countries with a RSV-F nanoparticle alum-adjuvanted vaccine showed that protection against RSV LRTI hospitalization was noted (44.4% vaccine efficacy, 95%CI: 19.6 to 61.5), but the primary study endpoint (per protocol analysis) for reduction of medically-significant RSV LRTI (39% vaccine efficacy; 97.5% CI: −1 to 63.7) was not met (albeit the 95% CI been 5.3 to 61.2) (224). This is the largest study so far to evaluate a vaccine primarily designed for use in pregnant women.

Multiple factors could have affected the outcomes measured in this first immunization study of a RSV vaccine in pregnancy. Pregnant women were vaccinated during 28–36 weeks gestation, and efficiency of transfer of anti-RSV antibodies were found to be higher in women vaccinated <30 weeks GA compared with women vaccinated ≥30 weeks GA. In addition, vaccine efficacy varied in different settings, being higher in middle-income countries (compared with HICs). Mathematical modeling can help predict women and infants who are expected to benefit the most from RSV vaccines. This could be achieved by defining women who are expected to deliver in RSV season and the preferred timing of vaccination to optimize protection in those infants. Ideal timing of vaccination could be predicted based on the kinetics of antibody response in mothers, the efficiency of antibody transfer and their estimated half-life, and duration of infants' exposure to seasonal RSV.

Based on the literature review and consultation among authors, a consensus on priorities for future research related to immunization during pregnancy against GBS and RSV was reached (Table 9).

**Table 9:** Consensus on priorities for future research related to vaccines against respiratory syncytial virus and Group B *Streptococcus* diseases during pregnancy.

**Group B *Streptococcus* vaccines**
1. The epidemiology of GBS disease in early life and risk factors for GBS disease in diverse geographic settings
2. Ideal composition of GBS vaccines to achieve highest protection against early and late onset GBS disease
3. Correlate(s) of protection against early and late onset GBS disease
4. Whether GBS vaccines given to pregnant women interfere with vaccines given in infancy and conjugated to TT and DT as carrier proteins
5. Effectiveness of GBS vaccines administered during pregnancy in reduction of early and late onset GBS disease
**Respiratory syncytial virus**
1. Definition of ideal timing of vaccination in pregnancy to achieve highest immunogenicity in infants
2. Mathematical modeling to inform clinical trials design to better define infants who will benefit the most from vaccination during pregnancy
3. Correlate(s) of protection against RSV disease in infancy
4. Epidemiology of RSV disease in 1st and 2nd years of life in offspring of mothers vaccinated during pregnancy

*GBS, Group B Streptococcus; TT, Tetanus toxoid; DT, Diphtheria toxoid; RSV, Respiratory Syncytial Virus*.

## Induction of Vaccine-Specific Immunity in Breastmilk

There is a paucity of information on the induction of antibodies in breastmilk following vaccination in pregnancy (225). Anti-*B. pertussis* secretory immunoglobulin A (sIgA) antibodies were detected in colostrum and in breast milk up to 8 weeks after delivery from women vaccinated with Tdap during pregnancy (226, 227). However, the clinical significance of these elevated *B. pertussis*-specific antibody concentrations in breastmilk has not been studied. A study from Bangladesh showed that vaccination with TIV in pregnancy induced influenza-specific sIgA levels in breastmilk for at least 6 months postpartum. In addition, breastfeeding was associated with a decrease in episodes of respiratory illness with fever in infants born to mothers vaccinated against influenza during pregnancy (228). A study from South Africa found that breastmilk sIgA against GBS was associated with lower risk for GBS LOD in young infants (229). In a study from Nepal, breastmilk RSV IgG levels, but not IgA levels, were lower in mothers of infants with RSV acute respiratory infection (230). While these studies report potential association between breastfeeding and reduction in disease risk, the casual relationship has not been confirmed.

Based on the literature review and consultation among authors, a consensus on priorities for future research related to the effect of immunization during pregnancy on the induction of vaccine-specific immunity in breast milk was reached (Table 10).

**Table 10:** Consensus on priorities for future research related to induction of vaccine-specific immunity in breast milk.

1. The additional role of breastfeeding in protection against clinical disease in infants born to mothers vaccinated against influenza, RSV and GBS during different phases of breastfeeding (colostrum, after 2–3 months of breastfeeding, etc.)
2. The additional role of breastfeeding in protection against clinical disease in infants born to mothers vaccinated against pertussis in settings where vaccine effectiveness is not optimal
3. Mechanisms of protection against respiratory pathogens through breastmilk

## Acceptance and Strategies for Increasing Uptake of Vaccines

The acceptance and coverage of immunization against tetanus during pregnancy in LMICs have been historically high (231). Despite recommendations by multiple health authorities worldwide, maternal immunization with influenza and pertussis vaccines has not been as widely accepted by healthcare workers or the general public, including pregnant women (232). Coverage remains suboptimal in many countries where recommendations for maternal immunization with influenza and pertussis vaccines have been in place for several years. In the US, influenza and pertussis vaccines have been recommended for all pregnant women since 2004 (233) and 2011 (234), respectively. However, during the 2017–2018 influenza season, only 49.1% of pregnant women received the influenza vaccine during the peak influenza season (235). During the same months, maternal Tdap uptake was only slightly higher (54.4%). Finally, the receipt of both vaccines was documented in only 32.8% of pregnant women (235). In the UK, where pertussis vaccine has been offered to all pregnant women since October 1, 2012, coverage during the period from April to June 2018 was 68.2% (236). In the European Union, although 90% of countries recommend vaccination against influenza for pregnant women, coverage was generally low in 2014–2015, with half of the countries reporting uptake of <10% (237). In France, during the 2015–16 season vaccine coverage was only 7.4% (238). In Switzerland influenza and pertussis immunizations in pregnancy have been recommended since 2009 and 2013, respectively. Yet, in a study performed in women who gave birth between 2013 and 2017, only five (3%) of 172 mothers had received both pertussis and influenza vaccines during pregnancy, 15 (9%) only against pertussis and 12 (7%) only against influenza (239).

Several factors can explain the poor compliance with the official recommendations. A recent literature review documented 25 individual patient-level and 24 healthcare provider-level barriers to maternal immunization uptake (240). Among the patient-identified barriers, concerns regarding safety for the mother or the newborn were very common and were cited in 6.4–71% and 2.9–77.0% of studies, respectively. Other vaccine and disease-related factors included concerns about vaccine efficacy, the belief that the disease was not sufficiently severe to require prevention, and the idea that healthy people did not need immunization. Moreover, several structural and logistical barriers were identified. Lack of insurance coverage, limited access or transportation, and the difficulty of finding a provider for vaccine administration were reported. Finally, social, psychological factors, and insufficient knowledge were listed repeatedly. Among the provider-level barriers, poor knowledge of the recommendations for immunization of pregnant women, financial concerns (inadequate reimbursement, payment, and/or complexity of billing), and inability to order, obtain and store vaccines. Globally, the lack of knowledge of vaccine recommendations seems to be the most important barrier for both health care workers (HCWs) (241–246) and pregnant women (247–252). HCWs in contact with pregnant women frequently have little experience in vaccines and therefore do not offer vaccinations to pregnant women (239, 253).

To overcome barriers to maternal immunization, both provider-focused and mother-focused interventions have been suggested, with a significant improvement in vaccine uptake has been evidenced in several cases (253). Suggested provider-focused interventions were notifying the provider of the vaccination status of pregnant women, establishing standing orders authorizing nursing staff to administer the vaccines without a medical consultation, giving provider feedback by reporting the vaccination rates of other institutions, and providing education to improve the knowledge and attitudes of HCWs toward vaccination in pregnancy. However, education of HCWs alone is probably ineffective if parental vaccine hesitancy is not addressed simultaneously.

Multiple educational efforts involving all HCWs who deliver care to pregnant women and the pregnant women themselves can yield positive results (254). This observation seems to have been confirmed by a study by Chamberlain et al., who showed that when obstetricians and women became familiar with the recommendation to promote and receive the influenza vaccine during pregnancy, the percentage of women who refused to be vaccinated declined from 88.9% in 2004 to 64.2% in 2011 (254). To overcome barriers in pregnant women, studies were planned to disseminate education and promotion of materials specifically for pregnant women by mass media campaigns via the internet, posters and leaflets, lectures and workshops, and personalized reminders and recall system (254). Integrating maternal immunization into routine obstetric care, with vaccine availability within the obstetrical setting, appears to be the best method of improving maternal immunization as well as subsequent childhood vaccine uptake (255).

Based on the literature review and consultation among authors, a consensus on priorities for future research related to acceptance and uptake of vaccines administered during pregnancy was reached (Table 11).

**Table 11:** Consensus on priorities for future research related to acceptance and strategies for increasing uptake of vaccines administered during pregnancy.

To identify strategies to increase tetanus vaccine coverage during pregnancy in low-middle income countries
The region and cultural specific approaches for implementation of vaccinations during pregnancy and their acceptance
The barriers to high maternal immunization uptake in specific populations.
The need of adequate education of health-care providers on maternal immunization and establishing a consensus on a minimum curriculum to be achieved during (para)medical education
The need of global information and awareness-raising campaigns
How to best inform pregnant women about new vaccines
The effectiveness of different strategies to increase influenza and pertussis vaccination coverage in pregnant women in different regions and cultures
To analyze similarities and differences in knowledge and attitudes to influenza and pertussis vaccination during pregnancy

## Conclusions

Pregnant women, their newborns and young infants are vulnerable to serious and potentially fatal infections. The new WHO goals aim to increase rates of live births and improve antenatal care for pregnant women (256), and vaccination in pregnancy is one strategy to improve health of pregnant women and their offspring. Safe and effective vaccines are already available against some diseases (tetanus, pertussis and influenza) for use during pregnancy, and these vaccines have the potential to prevent significant infectious disease morbidity and mortality in both the mothers and their offspring. In addition, new vaccines (e.g., RSV, GBS) are currently under development and are being tested in clinical trials, to be licensed and used in pregnant women. Following literature review and a consultation amongst experts in the fields of infectious diseases, vaccination and immunization during pregnancy, several gaps in knowledge and priorities for research were identified and are proposed (Tables 3–11). Addressing these priorities in future research has the potential to increase our understanding in different aspects of immunization during pregnancy and optimize protection for both the mother and the infant.

## Author Contributions

SE proposed the project, coordinated the study group, and wrote the first draft of the manuscript. BA-R wrote the first draft of the consensus statements and revised the initial draft of the manuscript. SO wrote the first draft of the ethics section. KM produced Figure 1. BA-R, KM, KE, SO, JE, MDS, GA, EL, PD, VP, OL, RD, MC, AC, KF, TF, SG, LV, MO'R, UH, NP, AA, MAS, NW, SM, MG, RP, SL, LM, FM-T, and SE reviewed and edited the manuscript, provided comments, suggested references, and substantially contributed to the content of the manuscript. BA-R, KM, KE, SO, JE, MDS, GA, EL, PD, VP, OL, RD, MC, AC, KF, TF, SG, LV, MO'R, UH, NP, AA, MAS, NW, SM, MG, RP, SL, LM, FM-T, and SE approved the final version of the manuscript. All authors contributed to the article and approved the submitted version.

## Conflict of Interest

SE: Research support from GSK, Sanofi, and Vifor. Speaker's fees from GSK, Pfizer, Novartis, Sanofi Pasteur, and MSD in the past 3 years. BA-R is supported by the Canadian Health and Research Institute Vanier Canada scholarship. KM is the beneficiary of a postdoctoral mandate fellowship from the FWO (Fund for Scientific Research-Flanders; FWO12R5819N). JE: Research support to my institution from Novavax, GlaxoSmithKline, Merck, Novavax, Chimerix; consultant for Sanofi Pasteur and Meissa. Have received both honoraria from both companies mentioned. MDS is currently, or has previously been, a Chief or Principal investigator on vaccine trials funded by vaccine manufacturers including GSK< MCM, Sanofi-Pasteur, Novartis Vaccines, Pfizer, Novavax, and Medimmune. These studies are conducted on behalf of the GSK, Novartis, Sanofi Pasteur MSD, and Novavax, University of Oxford and MDS receives no personal payment for this work. LV has received speaker's fees from GSK, Pfizer, Novartis, Sanofi Pasteur, and MSD. GlaxoSmithKline in the past 3 years. MO'R: Funding for clinical trials on Rotavirus Vaccines (GlaxoSmithKline up to 2008), Meningococcal b vaccines (GSK and Novartis up to 2017), RSV (Medimmue up to 2018), Pneumococcal vaccines (Merck to date). Funding for epidemiology/impact of disease studies: Enteric virus impact studies (Takeda vaccines to date), Vaccination acceptance study (Sanofi Pasteur to date). Travel support to present study results received; No speakers fees nor honorariums perceived. MAS has received grants to support research projects and consultancy fee from GSK, Pfizer, MSD, Seqirus, and Sanofi Pasteur. KF has served on the vaccine advisory boards for Sanofi-Pasteur and Seqiris in the past 3 years and received honoraria for attending meetings and speaker fees. SM; Institution received grant support in relation to studies on GBS and RSV including from BMGF, Pfizer, GSK, Minervax, and Novavax. No personal fees received from any of these sources, expect advisory honorarium from BMGF. FM-T has received honoraria from GSK, Pfizer, Sanofi Pasteur, Merck Sharp & Dohme, Seqirus, and Janssen for taking part in advisory boards and expert meetings, and for acting as speaker in congresses outside the scope of the submitted work. FM-T has also acted as principal investigator in RCTs of the above-mentioned companies as well as Ablynx, Regeneron, Roche, Abbot, Novavax, and Medimmune, with honoraria paid to his institution. FM-T research activities received support from the Instituto de Salud Carlos III (Proyecto de Investigación en Salud, Acción Estratégica en Salud): project ReSVinext ISCIII/PI16/01569/Cofinanciado FEDER and project Enterogen (ISCIII/PI19/01090). LM has received speaker's fees from GSK, Pfizer, Novartis, Sanofi Pasteur and MSD. RD has received grants/research support from Pfizer and Merck Sharp & Dohme; has been a scientific consultant for MeMed, Merck Sharp & Dohme, and Pfizer and a speaker for Pfizer. MC has received honoraria from GSK, Pfizer, Sanofi Pasteur, Merck Sharp & Dohme and Seqirus for taking part in advisory boards and expert meetings, and for acting as speaker in congresses outside the scope of the submitted work. MC has also been the principal investigator in RCTs of GSK, Sanofi Pasteur, and Novavax with honoraria paid to his institution. PD is (principal) investigator of vaccine trials for a large number of vaccine manufacturers (GSK Vaccines, Janssen Vaccines, Pfizer, Osyvax, Merck, Sanofi, and MSD) and institutions (Bill & Melinda Gates Foundation, EU-IMI, and FWO) for which the university of Antwerp obtains grants. UH is a member of the Global Pertussis Initiative (supported by Sanofi Pasteur, USA) and the Collaboration of European Experts on Pertussis Awareness Generation, CEEPAG (supported by Sanofi, France). The remaining authors declare that the research was conducted in the absence of any commercial or financial relationships that could be construed as a potential conflict of interest.
